# Coilin as a regulator of NF-kB mediated inflammation in preeclampsia

**DOI:** 10.1242/bio.059326

**Published:** 2022-07-25

**Authors:** Madelyn K. Logan, Katheryn E. Lett, Douglas M. McLaurin, Michael D. Hebert

**Affiliations:** Department of Cell and Molecular Biology, The University of Mississippi Medical Center, Jackson, MS 39216-4505, USA

**Keywords:** Coilin, Cajal body, MiR-517-3p, SFlt-1, Preeclampsia

## Abstract

The nuclear factor-Kappa B (NF-κB) pathway is a crucial mediator of inflammatory signaling. Aberrant activation of NF-κB is associated with several disorders including preeclampsia (PE). Many regulators of the NF-κB pathway have been identified, including microRNAs (miRNAs). Specifically, miR-517-3p targets mRNA encoding TNFAIP3 Interacting Protein 1 (TNIP1), an inhibitor of NF-κB signaling. Activation of NF-κB increases production of the cytokine TNF superfamily member 15 (TNFSF15), leading to the upregulation of anti-angiogenic soluble vascular endothelial growth factor receptor 1 (sFlt-1). We have previously observed that Cajal bodies (CBs), subnuclear domains, are associated with the chromosome 19 miRNA gene cluster (C19MC), which encodes miR-517-3p. We have also found that coilin, the CB marker protein, is a positive regulator of miRNA biogenesis. Here we report that coilin is a regulator of miR-517-3p, sFlt-1, TNIP1, TNFSF15 and NF-κB activation, and this regulation is influenced by hypoxia. We also report that coilin and CBs are induced in the reduced uterine perfusion pressure (RUPP) rat model of PE. Collectively, the data presented here implicate coilin as a novel regulator of NF-κB activation and sFlt-1 upregulation.

## Abstract (precis)

**Summary:** Coilin acts as an upstream regulator of the NF-kB pathway through positive regulation of miR-517-3p leading to increased NF-kB signaling and higher expression of sFlt-1.

## INTRODUCTION

The NF-κB family of proteins regulate multiple pathways that are involved in placental development (Cummins et al., 2007; Lingappan, 2018; Liu et al., 2017; Torchinsky and Toder, 2004; Zhou et al., 2003). Abnormal placental development can lead to pregnancy complications, such as preeclampsia (PE). During PE, the placenta is exposed to extreme oxidative stress and inflammation, accompanied by increased secretion of anti-angiogenic proteins compared to healthy control patients (Aouache et al., 2018; Chiarello et al., 2020; Sánchez-Aranguren et al., 2014). Inflammation, oxidative stress, and hypoxia can increase NF-κB activity (Culver et al., 2010; Koong et al., 1994; Melvin et al., 2011). Women with PE exhibit up to a tenfold increase in NF-κB expression in the placental and maternal circulation compared to normal pregnancies (Litang et al., 2017; Vaughan and Walsh, 2012). Increased activation of NF-κB in PE gives rise to the inappropriate secretion of anti-angiogenic proteins into the maternal-fetal circulation, which largely contributes to the maternal vascular dysfunction, proteinuria, and hypertension (Harmon et al., 2016; O'Brien et al., 2017; Rajakumar et al., 2009).

In recent years, microRNAs (miRNAs) have emerged as important regulators of gene expression in inflammatory and immune responses (Boldin and Baltimore, 2012; Taganov et al., 2007). miRNAs are small, non-coding RNAs that regulate gene expression primarily at the post-transcriptional level by acting as negative regulators of mRNA translation and stability, resulting in the suppression of translation. Multiple miRNAs alter NF-κB activity, either by targeting upstream NF-κB activating kinases or other NF-κB signaling components (Boldin and Baltimore, 2012). One potent activator of NF-κB signaling is miR-517-3p, which targets mRNA encoding the TNFAIP3 Interacting Protein 1 (TNIP1), an inhibitor of NF-κB signaling (Olarerin-George et al., 2013). Increased levels of miR-517-3p have also been found in placenta of women with PE. Increased NF-κB, as well as miR-517-3p, have both been found to lead to decreased trophoblast invasion and increased sFlt-1 release (Aban et al., 2004; Anton et al., 2015; Parrish et al., 2010; Shah and Walsh, 2007). This regulatory axis leading to the upregulation of sFlt-1 is thought to involve TNFSF15, a NF-κB induced gene that promotes alternative splicing of the *FLT1* gene in favor of sFlt-1 production.

We have previously identified the Cajal bodies (CBs) marker protein, coilin, as a novel regulator of miRNA biogenesis (Lett et al., 2021; Logan et al., 2021, 2020). CBs are phase-separated nuclear condensates that contribute to the biogenesis and maturation of ribonucleoproteins (RNPs). CB nucleation increases in hypoxic conditions and they can spatially form at, or be recruited to, non-coding RNA transcribed loci including the placenta specific C19MC where miR-517-3p is expressed (Logan et al., 2020). This suggests CBs could be an upstream regulator of the miR-517-3p pathway that involves NF-κB and leads to the upregulation of sFlt-1. Here we present data demonstrating that coilin and CBs are novel regulators of NF-κB activation and sFlt-1 upregulation in JEG-3 choriocarcinoma cells. We also show that coilin impacts miR-517-3p, TNIP1 and TNFSF15 levels. Additionally, we report here that coilin and CBs are upregulated in RUPP rat placenta, further supporting our hypothesis that coilin/CBs may operate through placentally expressed miRNA gene clusters to contribute to the abnormal placentation observed in PE.

## RESULTS and DISCUSSION

### Coilin and CBs are induced in the RUPP rat model of PE

**Fig. 1.**
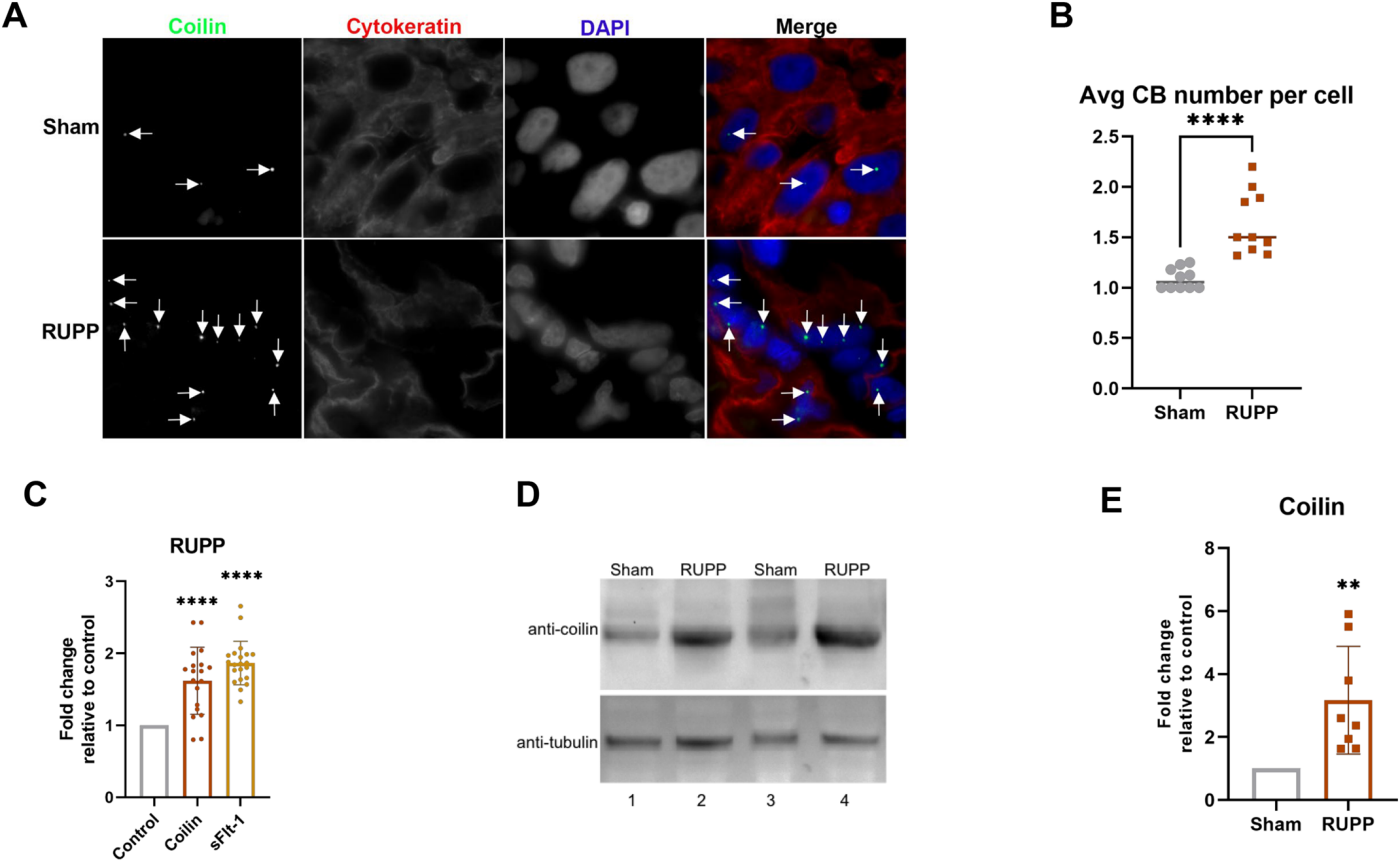
**Coilin and CBs are induced in placenta from the RUPP rat.** (A) Placental tissue from Sham and RUPP rats were fixed followed by IHC detection of coilin (green), cytokeratin (a marker for trophoblasts, red) and DAPI staining for nuclei (blue). Arrows denote CBs. (B) Histogram depicting average number of CBs per cell in Sham and RUPP (*N*=275). (C) Coilin and sFlt-1 mRNA quantification in RUPP placenta tissue normalized to GAPDH relative to that detected in Sham placenta, which is set to 1. *N*=18 including six biological repeats. (D) Western blot of lysate from Sham and RUPP placental tissue, probed for coilin and β-tubulin. (E) Quantification of coilin/tubulin ratio in RUPP placenta, relative to that in Sham placenta, which is set to 1. *N*=8 biologicals. For all panels with statistics, error bars represent standard deviation and **=*P*<0.005, ****=*P*<0.0001.

Our previous work demonstrates a hypoxia-dependent induction of CB nucleation in primary fibroblasts (Logan et al., 2021). Other work has found CB induction in placenta from women with severe PE (Gormley et al., 2017). In support of these findings, we hypothesized a clinical hypoxic model of PE could also induce CBs. For these experiments, we used the well-characterized clinically relevant RUPP rat model of PE. The RUPP procedure as described (Crews et al., 2000) decreases uterine blood flow by approximately 40%. Immunohistochemistry of placental slices from control (sham) or RUPP rats showed a significant increase in the average number of CBs per cell in RUPP trophoblasts (Fig. 1A,B). While there was an increase in the number of CBs, we did not find any significant difference in the relative size or intensity of coilin fluorescence per CB in RUPP trophoblasts compared to Sham (Fig. S1A). Interestingly, lysate from RUPP total placenta contains more coilin mRNA (Fig. 1C) and coilin protein (Fig. 1D,E) compared to sham lysate. This is consistent with our previously published data showing that primary fibroblasts treated with hypoxia also had an increase in coilin and CBs, and further verifies hypoxia as a regulator of CB formation (Logan et al., 2021).

### CB proteins influence miR-517-3p and sFlt-1 mRNA levels

**Fig. 2.**
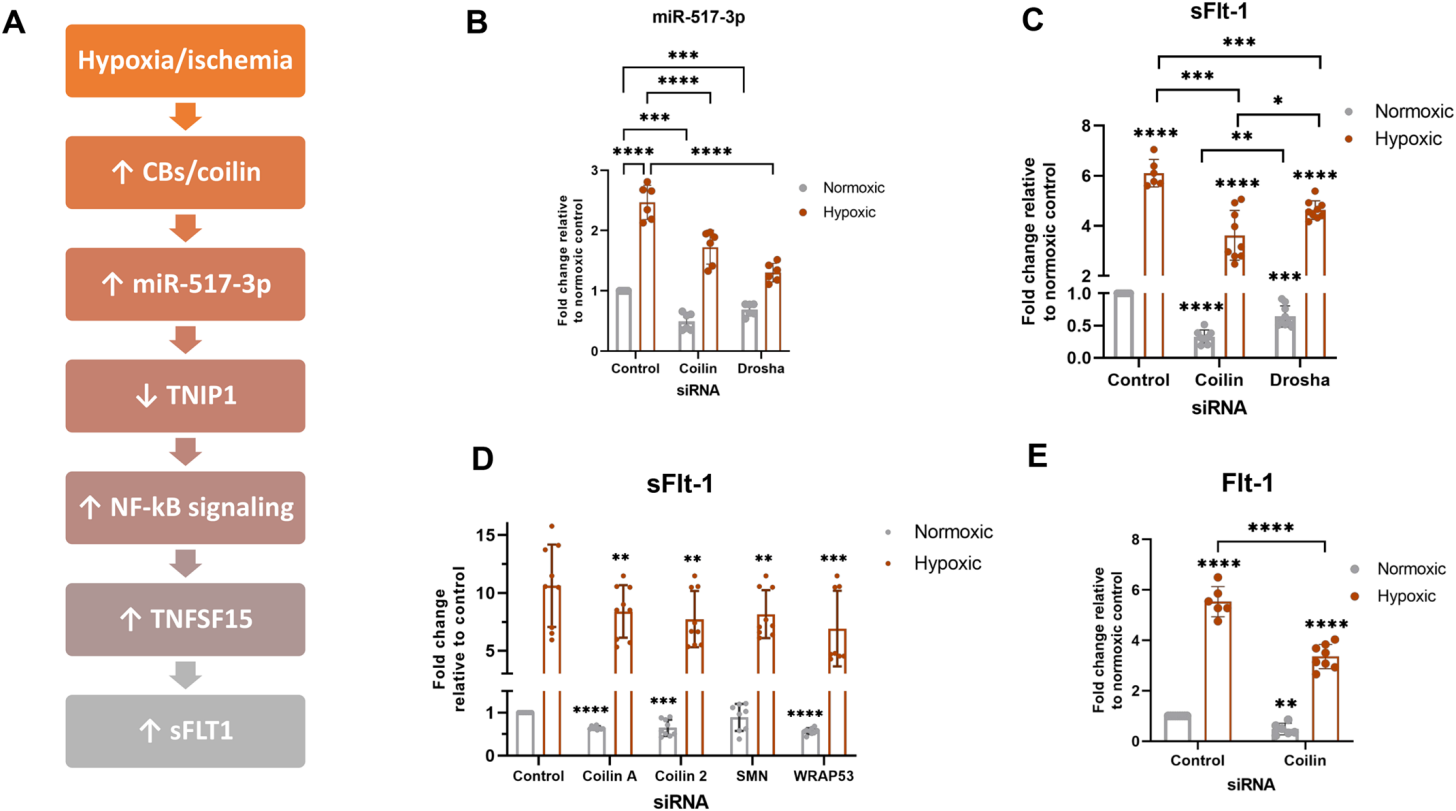
**CB protein regulation of miR-517-3p and sFlt-1.** (A) Schematic of the hypothesis tested linking the pathway from CBs to increased sFlt-1 in hypoxia. (B–E) JEG-3 cells were transfected with negative control or the indicated siRNAs for 24 h followed by 48 h normoxic (21% O_2_) or hypoxic (1% O_2_) treatment. (B) miR-517-3p was quantified by qPCR with 5S rRNA used as the normalizer. sFlt-1 (C,D) and Flt-1 (E) were quantified by qRT-PCR with β-actin mRNA used as the normalizer. Data are shown normalized to the normoxic control, which is set to 1. Significance is represented compared to the respective normoxic or hypoxic control, unless otherwise specified. *N*=at least six including three biological repeats. Error bars represent standard deviation and *=*P*<0.05, **=*P*<0.005, ***=*P*<0.0002, ****=*P*<0.0001.

We have previously published that the reduction of proteins enriched in the CB decreases miRNA biogenesis (Lett et al., 2021; Logan et al., 2021, 2020). Importantly, we have also published that coilin reduction in primary cell lines that have few CBs, such as WI-38, also results in decreased miRNA levels, suggesting that coilin contributes to miRNA biogenesis in cells with or without CBs (Lett et al., 2021; Logan et al., 2021, 2020). Therefore, we hypothesize CBs could be an upstream regulator of the miR-517-3p pathway that involves NF-κB and leads to the upregulation of sFlt-1 in hypoxia (Fig. 2A). Descriptions of the components of this pathway and their roles in PE are summarized in Table 1. To determine if coilin can regulate miR-517-3p biogenesis in normoxic and hypoxic conditions, we used JEG-3 choriocarcinoma cells due to miR-517-3p residing in the placentally expressed C19MC cluster. For this analysis, cells were transfected with coilin siRNA or Drosha siRNA for 24 h followed by 48 h normoxic (21% O_2_) or hypoxic (1% O_2_) incubation and subsequent qPCR detection of miR-517-3p. Drosha is an important miRNA biogenesis component and Drosha KD, therefore, is a positive control for altered miRNA processing that does not affect CBs while coilin KD abolishes canonical CBs (Lemm et al., 2006). As shown in Fig. 2B, the relative level of miR-517-3p is significantly decreased upon coilin KD in normoxic conditions. In hypoxic conditions with control siRNA, miR-517-3p is induced compared to that observed with normoxia, in agreement with previous studies using PE placental tissue (Anton et al., 2015). The induction of miR-517-3p in hypoxia is blunted with coilin KD. As expected, Drosha KD also results in a decrease of miR-517-3p in both normoxia and hypoxia.

**Table 1.**
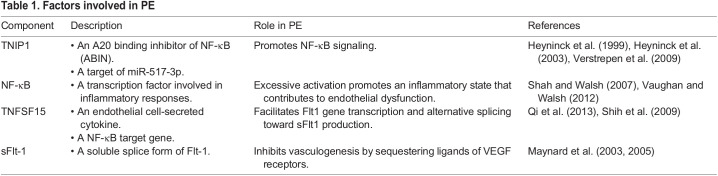
Factors involved in PE

We showed previously that CBs functionally associate with C19MC at a percentage equivalent to known CB association with non-coding RNA (e.g. *RNU2*) loci (Logan et al., 2020). We followed up those studies by investigating the CB/C19MC association frequency with hypoxia and found no statistical significance in the association frequency between normoxic and hypoxic treated JEG-3 cells.

Because miR-517-3p has been linked to sFlt-1 induction (Anton et al., 2015), we next investigated if coilin KD impacts sFlt-1 mRNA levels in normoxia and hypoxia. As expected, sFlt-1 is induced by hypoxia in control siRNA treated cells (Fig. 2C). Similar to what was observed with miR-517-3p, coilin KD decreased sFlt-1 levels in normoxic conditions and attenuated the induction of sFlt-1 by hypoxia. We also found that Drosha KD in normoxia and hypoxia likewise reduces sFlt-1 mRNA levels, further implicating that disrupted miRNA biogenesis takes place upon coilin KD. A similar profile of altered sFlt-1 mRNA in normoxia and hypoxia was observed using an additional coilin siRNA as well as KD of the CB-enriched SMN and WRAP53 proteins (Fig. 2D), supporting our previous data implicating CBs as miRNA regulators (Logan et al., 2021, 2020). Interestingly, the full-length membrane bound isoform of the *FLT1* gene, Flt-1, is also decreased with coilin KD (Fig. 2E). This suggests that coilin may be acting as a regulator of sFlt-1 alternative splicing through the miR-517-3p pathway, but also could be regulating the stability of Flt-1 mRNA from which sFlt-1 is alternatively spliced.

### Coilin is a negative regulator of NF-κB activation

**Fig. 3.**
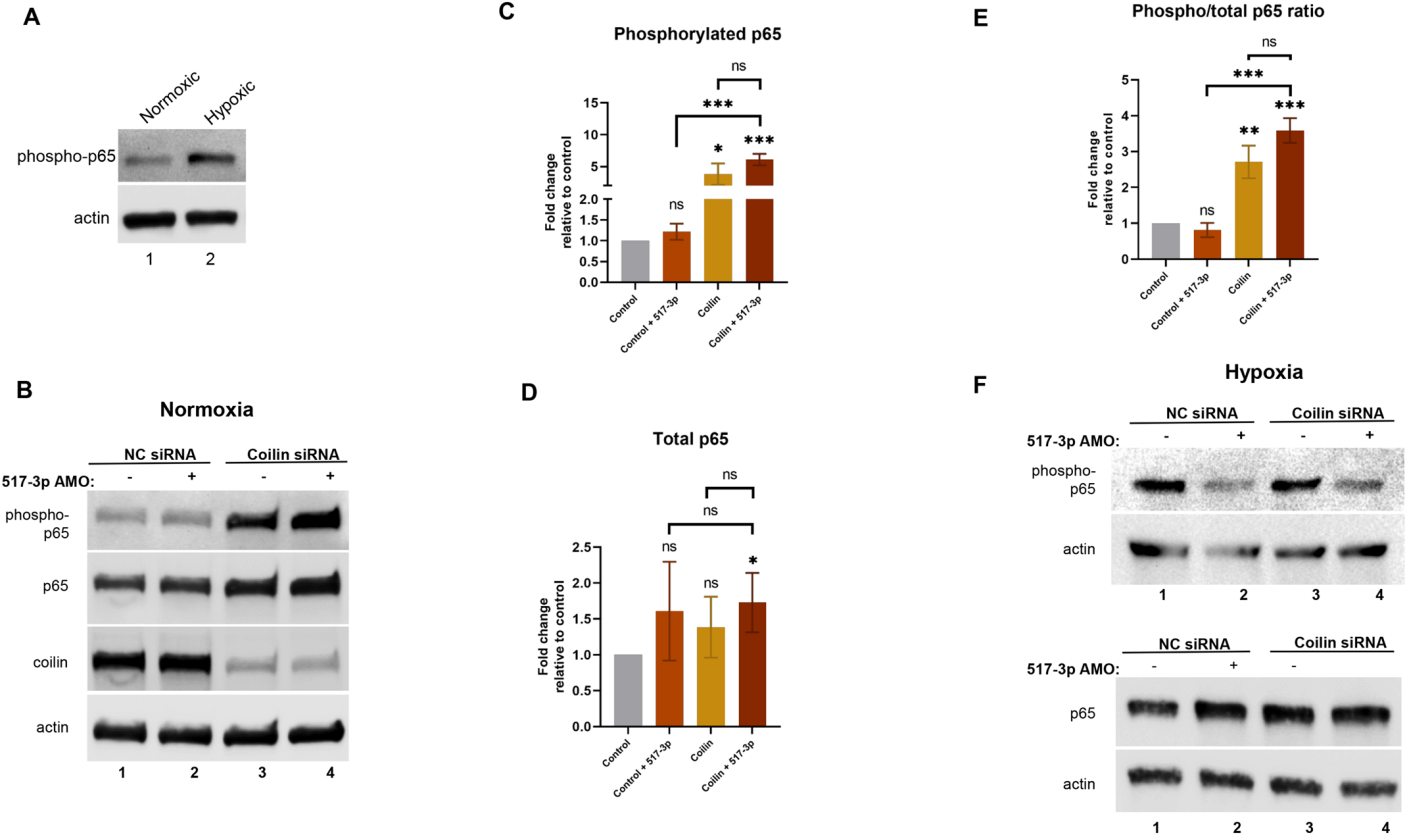
**Coilin negatively regulates NF-κB in normoxia.** (A) JEG-3 cells were incubated in normoxic or hypoxic conditions for 48 h, followed by protein detection of phosphorylated p65 and actin. (B) JEG-3 cells were transfected for 72 h with negative control siRNA (lanes 1 and 2) or coilin siRNA (lanes 3 and 4). Lanes 2 and 4 were additionally transfected with a miR-517-3p anti-miRNA oligo. Lysates were subjected to western blotting and the same membrane was sequentially probed with a mouse antibody to phosphorylated p65, a rabbit antibody to total p65, a rabbit antibody to coilin, and a mouse antibody to actin. (C,D) Quantification of western blot signals for phosphorylated p65 (C) and total p65 (D), relative to actin and normalized to the control siRNA signal, which is set to 1. (E) Histogram depicting the ratio of phosphorylated p65 to total p65 from the quantified histograms. *N*=3 biologicals. For all panels with statistics, error bars represent standard deviation and *=*P*<0.05, **=*P*<0.005, ***=*P*<0.0002. (F) same as B, except cells were treated with control or coilin siRNA and miR-517-3p AMO for 24 h followed by 48 h hypoxia.

The canonical signaling pathway of NF-κB involves phosphorylation, polyubiquitination and degradation of the IκBα sequestering complex, allowing bound NF-κB dimers to translocate to the nucleus and activate gene expression. Subsequent to nuclear translocation, the RelA (p65) monomer undergoes phosphorylation to further enhance NF-κB function as a transcription factor (Bohuslav et al., 2004; Madrid et al., 2001; Sakurai et al., 1999; Sasaki et al., 2005; Song et al., 2006; Takeuchi et al., 2013). Since miR-517-3p activates NF-κB by decreasing the level of the inhibitor TNIP1, and coilin KD reduces miR-517-3p levels (Fig. 2), we next examined if coilin can impact NF-κB activation. We also examined NF-κB activation in the presence of an anti-miRNA oligonucleotide (AMO) to miR-517-3p, alone or in combination with coilin KD, with and without hypoxia. Activation of NF-κB was assessed by monitoring the level of p65 ser536 phosphorylation by western blot. As shown in Fig. 3A, and in agreement with previous reports (Culver et al., 2010; Koong et al., 1994; Melvin et al., 2011), hypoxia increases p65 ser536 phosphorylation. Very interestingly, coilin KD in normoxic conditions induces NF-κB as detected by p65 ser536 phosphorylation (Fig. 3B, compare top panel signal in lane 3 to that in lane 1). Inhibition of miR-517-3p did not alter the level of p65 ser536 phosphorylation, with or without coilin KD. The same blot was reprobed with an antibody to total p65, coilin (to verify coilin KD) and actin. Quantification of this and other blots shows that coilin KD significantly increases the amount of p65 ser536 phosphorylation relative to total p65 (Fig. 3E) and this induction was not affected by miR-517-3p inhibition. The increase in p65 phosphorylation upon coilin KD was also observed with an additional coilin siRNA (Fig. S2A). Additionally, increased p65 phosphorylation was seen with SMN knockdown, but not WRAP53 knockdown (Fig. S1C–E).

While the increased p65 ser536 phosphorylation seen with coilin knockdown suggests that coilin is a negative regulator of NF-κB activation in normoxia instead of a positive regulator, there could be non-canonical pathways leading to NF-κB activation. In one such pathway, DNA damage activates NF-κB by a mechanism involving the serine/threonine kinase ribosomal S6 kinase 1 (RSK1) stimulation, which in turn phosphorylates p65 at ser536. Several studies have found coilin participates in the nonhomologous end-joining (NHEJ) pathway of DNA damage repair (Bartova et al., 2014; Velma et al., 2010). Knocking down coilin could increase RSK1 resulting in activation of the non-canonical NF-κB pathway. We tested this hypothesis but did not observe an increase in RSK1 with coilin KD (Fig. S1F,G). Alternatively, human coilin-interacting nuclear ATPase protein (hCINAP), a binding partner to coilin's C terminus (Santama et al., 2005), negatively regulates NF-κB signaling by interacting with the IKK complex and inhibiting IKK phosphorylation (Qu et al., 2015). Even though our data suggest coilin is a negative regulator of NF-κB activation, however, coilin and miR-517-3p could still be positive regulators in hypoxia. Hence, we examined the level of p65 ser536 phosphorylation upon coilin KD and miR-517-3p inhibition in hypoxic conditions and we observed that miR-517-3p is required for NF-κB activation (Fig. 3F). In contrast, coilin KD in hypoxia did not alter the level of p65 ser536 phosphorylation, with or without co-treatment with miR-517-3p AMO, compared to that observed with control siRNA. Therefore, the direct relationship between coilin knockdown and phosphorylated p65 in hypoxia may include additional regulators not including mir-517-3p. However, these findings suggest that, in JEG-3 hypoxic cells, the miR-517-3p::TNIP1::NF-κB activation regulatory axis is still functional with miR-517-3p.

### Differential effects of coilin on TNIP1 and TNFSF15 mRNA levels

**Fig. 4.**
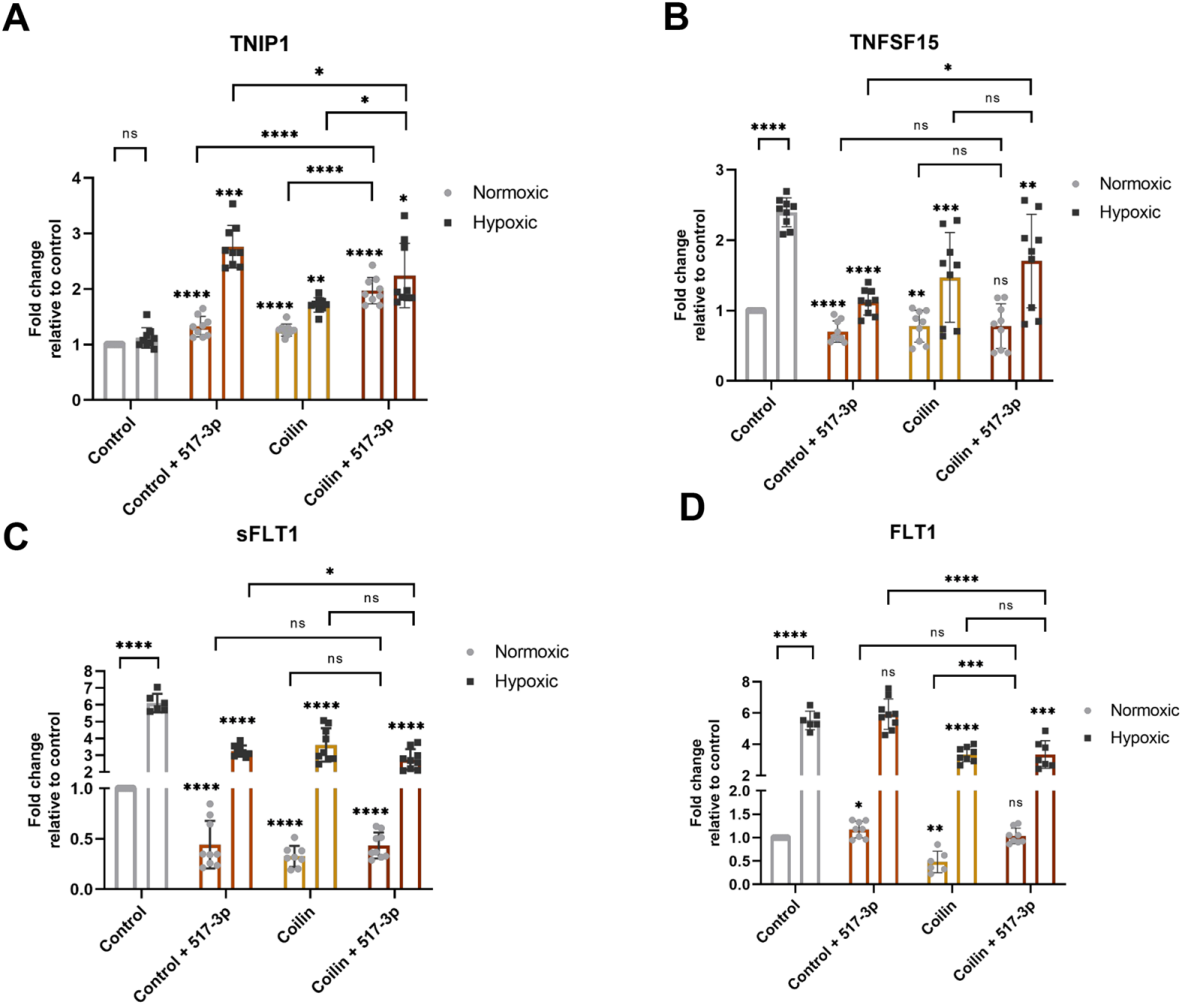
**Dysregulation of TNIP1, TNFSF15, sFlt-1 and Flt-1 mRNA upon coilin knockdown and miR-517-3p inhibition.** JEG-3 cells were transfected with siRNA or co-transfected with siRNA plus miR-517-3p AMO for 24 h followed by 48 h normoxic or hypoxic treatment. TNIP1 (A), TNFSF15 (B), sFlt-1 (C), and Flt-1 (D) mRNA levels were quantified by qRT-PCR. β-actin mRNA was used as the normalizer and data are shown relative to the control siRNA normoxic condition, which is set to 1. Significance is represented compared to the respective normoxic or hypoxic control, unless otherwise specified. *N*=at least six including three biological repeats. For all panels with statistics, error bars represent standard deviation and *=*P*<0.05, **=*P*<0.005, ***=*P*<0.0002, ****=*P*<0.0001.

TNIP1 inhibits NF-κB activity by facilitating proteasome degradation of upstream regulators (Heyninck et al., 1999; Nanda et al., 2011). Because we found coilin KD decreases miR-517-3p (Fig. 2B), which targets TNIP1 mRNA, we next examined if coilin KD would alter the regulation of TNIP1 by miR-517-3p and hypoxia. Specifically, we found that coilin KD slightly but significantly increases TNIP1 mRNA levels in both normoxic and hypoxic conditions compared to control siRNA treated cells (Fig. 4A). Simultaneous knockdown of coilin and miR-517-3p further increases TNIP1 mRNA, but the highest levels of TNIP1 mRNA were obtained in hypoxic cells treated with the miR-517-3p AMO. These findings indicate that TNIP1 expression is most sensitive to miR-517-3p regulation during hypoxia.

We next examined the level of a NF-κB induced gene, *TNFSF15*, upon coilin, miR-517-3p AMO and hypoxia treatment. TNFSF15 was initially identified as a specific inhibitor of angiogenesis (Yue et al., 1999; Zhai et al., 1999), and we have found that TNFSF15 mRNA levels are induced by hypoxia in JEG-3 choriocarcinoma cells (Fig. 4B). This finding is consistent with a regulatory axis wherein hypoxic induction of miR-517-3p decreases TNIP1 levels, resulting in increased NF-κB activation and subsequent upregulation of TNFSF15 and sFlt-1 levels. In support of this idea, inhibition of miR-517-3p diminishes TNFSF15 induction in the presence of hypoxia (Fig. 4B). Coilin KD with hypoxia likewise blunts the induction of TNFSF15 mRNA levels compared to control siRNA treated hypoxic cells. Coilin KD coupled with miR-517-3p inhibition during hypoxia leads to similar reductions in TNFSF15 mRNA levels as found with just coilin KD.

Due to TNFSF15's function of promoting Flt-1 gene transcription, and alternative splicing of Flt-1 mRNA in favor of sFlt-1 (Endo et al., 2010; Qi et al., 2013), decreased TNFSF15 levels resulting from coilin KD in both normoxic and hypoxic conditions should decrease the sFlt-1/Flt-1 ratio. As Fig. 4C and D show, sFlt-1 and Flt-1 mRNA levels are both significantly decreased in normoxic and hypoxic samples with coilin knockdown compared to that observed with control siRNA. miR-517-3p inhibition also decreases sFlt-1 levels in normoxic and hypoxic conditions (Fig. 4C). In contrast with coilin KD however, miR-517-3p inhibition slightly increases Flt-1 in normoxia and does not change Flt-1 in hypoxia in the control siRNA background (Fig. 4D). These findings suggest that miR-517-3p and coilin may operate through similar pathways to regulate sFlt-1, but coilin also is a positive regulator of Flt-1. In agreement with this supposition, simultaneous reduction of coilin and miR-517-3p does not alter the level of sFlt-1 or Flt-1 observed with coilin KD alone. However, we do observe a slight increase in Flt-1 levels in normoxic cells in which both coilin and miR-517-3p are reduced compared to that observed with coilin KD alone (Fig. 4D).

In conclusion, the work presented here provides evidence supporting coilin's role in miRNA regulation and healthy placentation. Importantly, our studies demonstrate coilin plays opposing roles in regulating NF-κB signaling depending on the presence of hypoxia or not. While the mechanism behind the activation of NF-κB in normoxia with coilin KD is still unclear, we show miR-517-3p is still positively regulated by coilin and this is the ultimate factor in decreasing NF-κB signaling in hypoxia. Future studies will aim to determine how NF-κB signaling is upregulated with coilin KD. While sFlt-1 is a notorious anti-angiogenic factor in PE, NF-κB signaling during pathological pregnancies induces expression of multiple other pro-inflammatory and anti-angiogenic proteins that further promote inflammation and vascular dysfunction (Armistead et al., 2020). Although there are novel therapeutics targeting NF-κB for PE women (Eddy et al., 2020), fully understanding the upstream contributors to activated NF-κB is necessary for PE disease characterization. Additionally, future studies will be needed to fully elucidate coilin's effect on downstream NF-κB promoted anti-angiogenic proteins, opening up a novel avenue of research to investigate the molecular mechanisms by which this occurs.

## MATERIALS AND METHODS

### Cell lines, treatments, and transfections

The JEG-3 cell line was obtained from the American Type Culture Collection (ATCC). Cells were cultured as previously described (Enwerem et al., 2014). All siRNAs and miRNA inhibitors were obtained from Integrated DNA Technologies (Coralville, IA, USA) and utilized with RNAiMAX (Invitrogen, Carlsbad, CA, USA) per the manufacturer's protocol. Negative control, coilin, Drosha, SMN, Coilin A, and Coilin 3′ UTR siRNAs have been previously described (Logan et al., 2018; Poole et al., 2016). All coilin knockdowns refer to Coilin 2 siRNA unless mentioned. miRNA inhibitors used were: NC: (5′- GCGUAUUAUAGCCGAUUAACG −3′), and miR-517-3p (5′- ACACUCUAAAGGGAUGCACGA −3′). siRNA and miRNA inhibitor transfections were conducted for 72 h. Hypoxia treatments were carried out as previously described (Logan et al., 2021).

### Animals

Timed-pregnant Sprague-Dawley rats obtained at gestation day 11 (Envigo, Indianapolis, IN, USA) were held at a constant 23°C, and were put on a 12:12-h light–dark cycle with food and water *ad libitum*. All protocols were approved by the University of Mississippi Medical Center Institutional Animal Care and Use Committee and followed the National Institutes of Health Guidelines for the Care and Use of Laboratory Animals.

### RUPP procedure

On gestational day 14, RUPP-treated rats were subjected to aortic and bilateral ovarian artery constriction as previously described (Alexander et al., 2001). Briefly, animals were anesthetized by controlled 3% isoflurane (Webster), and a midline abdominal incision was made. After externalization of both uterine horns, one single 0.203 mm silver surgical clip was placed on the abdominal aorta above the iliac bifurcation. One 0.100 mm silver surgical clip was placed on both the left and right ovarian arteries, which supply the uterus, to prevent compensatory flow. Sham, control-treated animals were incised, but no clips were placed.

### Tissue harvest

Rats were anesthetized as above. The uterus was externalized, through a ventral midline incision, and placental samples (one from each) were flash frozen in liquid nitrogen. 0.15 g of placental tissue were removed and rinsed in cold 1X PBS and lysed in 1X RIPA+PIC buffer using the FastPrep (MP Biomedicals, Irvine, CA, USA) homogenization method. Samples were further sonicated and then centrifuged at 13,200 rpm for 15 min at 4°C. We acknowledge both the George and Reckelhoff labs (UMMC) for providing the placental tissue.

### Western blotting

JEG-3 cells were lysed in RIPA and lysate was run on a precast 10% Mini-Protean Gel (Bio-Rad Laboratories, Hercules, CA, USA) as previously described (Poole et al., 2016). Western transfer and detection was then conducted using the Trans-Blot Turbo Transfer System and SuperSignal West Pico Chemiluminescent Substrate (Bio-Rad Laboratories, Hercules, CA, USA) according to the manufacturer's protocol. The primary antibodies used were: anti-phospho-NF-κB p65 rabbit monoclonal antibody (93H1, Cell Signaling Technology, Danvers, MA, USA), anti-NF-κB p65 mouse monoclonal (L8F6, Cell Signaling Technology, Danvers, MA, USA), anti-coilin rabbit polyclonal antibody (H-300, Santa Cruz Biotechnology Inc., Dallas, TX, USA), anti-Drosha rabbit monoclonal antibody (D28B1, Cell Signaling Technology, Danvers, MA, USA), and anti-β-actin mouse monoclonal antibody (8H10D10, Cell Signaling Technology, Danvers, MA, USA). Secondary antibodies used were goat anti-mouse HRP and goat anti-rabbit HRP. Band detection and image adjustments were done as previously described (Poole et al., 2016).

### Quantitative real-time PCR

RNA was extracted from JEG-3 cells or rat placenta tissue with TRI-REAGENT (Molecular Research Center, Cincinnati, OH, USA) according to the manufacturer's suggested protocol. Reactions were set up as previously described (Logan et al., 2021). Oligonucleotides used were obtained from Integrated DNA Technologies (Coralville, IA, USA) with β-actin, coilin, and Drosha, as previously described (Burke et al., 2019). All other primer sequences can be found in Table S1. For RT-PCR detection of miRNA, the miRCURY LNA RT and PCR kits (Qiagen, Germantown, MD, USA) were used according to the manufacturer's protocol. Primers for 5 s rRNA and all miRNAs were obtained from the manufacturer (Qiagen, Germantown, MD, USA).

### Immunohistochemistry (IHC)

Formalin fixed control (Sham) and RUPP placenta paraffin embedded blocks obtained from the Alexander lab were cut at 4 μm on a microtome and mounted on Superfrost Plus slides (Thermo Fisher Scientific Inc., Waltham, MA, USA). Immunofluorescence staining was done following the Zaqout et al. protocol (Zaqout et al., 2020). Briefly, slides were dewaxed using xylene and a 100, 95, and 70% ethanol series. The unmasking step was done using a vegetable steamer and preheated 10 mM sodium citrate solution at pH 6. Permeabilization was conducted using a 0.25% Triton solution. Slides were then blocked with 5% NGS and then incubated with a 1:200 primary antibody solution at room temperature overnight in a humidifying chamber. Trophoblast cells were detected with anti-cytokeratin pan mouse monoclonal antibody (C-11, Invitrogen, Waltham, MA, USA) while CBs were detected with anti-coilin rabbit polyclonal antibody (H-300, Santa Cruz Biotechnology Inc., Dallas, TX, USA). Slides were then washed with 1X PBS and 0.25% Triton and incubated with a secondary antibody solution containing: 1:600 Alexa Fluor 488 (A11008, Invitrogen, Carlsbad, CA, USA) goat anti-rabbit (green), 1:600 Alexa Fluor 594 (A32742, Invitrogen, Carlsbad, CA, USA) goat anti-mouse (red) secondary antibody, and 1:100 DAPI in 10% NGS at room temperature. Slides were then washed in 1X PBS and 10 mM copper sulfate/50 mM ammonium chloride solution to reduce background signal followed by coverslip mounting with Antifade (Invitrogen, Carlsbad, CA, USA). Cells were imaged as previously described (Logan et al., 2020; Poole et al., 2016). For statistical analysis, ten images of each group (Sham and RUPP) were blindly captured by scanning in the DAPI filter. Each image was scored for total number of CBs expressed per cell. The individual numbers were then combined for all ten images and the total percent of cells expressing CBs in each group was calculated.

## Supplementary Material

Supplementary information

## References

[BIO059326C1] Aban, M., Cinel, L., Arslan, M., Dilek, U., Kaplanoglu, M., Arpaci, R. and Dilek, S. (2004). Expression of nuclear factor-kappa B and placental apoptosis in pregnancies complicated with intrauterine growth restriction and preeclampsia: an immunohistochemical study. *Tohoku J. Exp. Med.* 204, 195-202. 10.1620/tjem.204.195 15502418

[BIO059326C2] Alexander, B. T., Cockrell, K., Cline, F. D., Llinas, M. T., Sedeek, M. and Granger, J. P. (2001). Effect of angiotensin II synthesis blockade on the hypertensive response to chronic reductions in uterine perfusion pressure in pregnant rats. *Hypertension* 38, 742-745. 10.1161/01.HYP.38.3.742 11566968

[BIO059326C3] Anton, L., Olarerin-George, A. O., Hogenesch, J. B. and Elovitz, M. A. (2015). Placental expression of miR-517a/b and miR-517c contributes to trophoblast dysfunction and preeclampsia. *PLoS One* 10, e0122707. 10.1371/journal.pone.0122707 25799546 PMC4370750

[BIO059326C4] Aouache, R., Biquard, L., Vaiman, D. and Miralles, F. (2018). Oxidative stress in preeclampsia and placental diseases. *Int. J. Mol. Sci.* 19, 1496. 10.3390/ijms19051496 PMC5983711 29772777

[BIO059326C5] Armistead, B., Kadam, L., Drewlo, S. and Kohan-Ghadr, H. R. (2020). The role of NFκB in healthy and preeclamptic placenta: trophoblasts in the spotlight. *Int. J. Mol. Sci.* 21, 1775. 10.3390/ijms21051775 PMC7084575 32150832

[BIO059326C6] Bartova, E., Foltankova, V., Legartova, S., Sehnalova, P., Sorokin, D. V., Suchankova, J. and Kozubek, S. (2014). Coilin is rapidly recruited to UVA-induced DNA lesions and γ-radiation affects localized movement of Cajal bodies. *Nucleus* 5, 269-277. 10.4161/nucl.29229 PMC4133222 24859326

[BIO059326C7] Bohuslav, J., Chen, L. F., Kwon, H., Mu, Y. and Greene, W. C. (2004). p53 induces NF-κB activation by an IκB kinase-independent mechanism involving phosphorylation of p65 by ribosomal S6 kinase 1. *J. Biol. Chem.* 279, 26115-26125. 10.1074/jbc.M313509200 15073170

[BIO059326C8] Boldin, M. P. and Baltimore, D. (2012). MicroRNAs, new effectors and regulators of NF-κB. *Immunol. Rev.* 246, 205-220. 10.1111/j.1600-065X.2011.01089.x 22435557

[BIO059326C9] Burke, M. F., McLaurin, D. M., Logan, M. K. and Hebert, M. D. (2019). Alteration of 28S rRNA 2'-O-methylation by etoposide correlates with decreased SMN phosphorylation and reduced Drosha levels. *Biol. Open* 8, bio041848. 10.1242/bio.041848 30858166 PMC6451326

[BIO059326C10] Chiarello, D. I., Abad, C., Rojas, D., Toledo, F., Vazquez, C. M., Mate, A., Sobrevia, L. and Marín, R. (2020). Oxidative stress: normal pregnancy versus preeclampsia. *Biochim. Biophys. Acta Mol. Basis Dis.* 1866, 165354. 10.1016/j.bbadis.2018.12.005 30590104

[BIO059326C11] Crews, J. K., Herrington, J. N., Granger, J. P. and Khalil, R. A. (2000). Decreased endothelium-dependent vascular relaxation during reduction of uterine perfusion pressure in pregnant rat. *Hypertension* 35, 367-372. 10.1161/01.HYP.35.1.367 10642326

[BIO059326C12] Culver, C., Sundqvist, A., Mudie, S., Melvin, A., Xirodimas, D. and Rocha, S. (2010). Mechanism of hypoxia-induced NF-κB. *Mol. Cell. Biol.* 30, 4901-4921. 10.1128/MCB.00409-10 20696840 PMC2950552

[BIO059326C13] Cummins, E. P., Comerford, K. M., Scholz, C., Bruning, U. and Taylor, C. T. (2007). Hypoxic regulation of NF-κB signaling. *Methods Enzymol.* 435, 479-492. 10.1016/S0076-6879(07)35025-8 17998070

[BIO059326C14] Eddy, A. C., Howell, J. A., Chapman, H., Taylor, E., Mahdi, F., George, E. M. and Bidwell, G. L., 3rd. (2020). Biopolymer-delivered, maternally sequestered NF-κB (nuclear factor-κB) inhibitory peptide for treatment of preeclampsia. *Hypertension* 75, 193-201. 10.1161/HYPERTENSIONAHA.119.13368 31786977 PMC7008946

[BIO059326C15] Endo, K., Kinouchi, Y., Kakuta, Y., Ueki, N., Takahashi, S. and Shimosegawa, T. (2010). Involvement of NF-kappa B pathway in TL1A gene expression induced by lipopolysaccharide. *Cytokine* 49, 215-220. 10.1016/j.cyto.2009.09.006 19815424

[BIO059326C16] Enwerem, I. I., Velma, V., Broome, H. J., Kuna, M., Begum, R. A. and Hebert, M. D. (2014). Coilin association with Box C/D scaRNA suggests a direct role for the Cajal body marker protein in scaRNP biogenesis. *Biol. Open* 3, 240-249. 10.1242/bio.20147443 24659245 PMC3988793

[BIO059326C17] Gormley, M., Ona, K., Kapidzic, M., Garrido-Gomez, T., Zdravkovic, T. and Fisher, S. J. (2017). Preeclampsia: novel insights from global RNA profiling of trophoblast subpopulations. *Am. J. Obstet. Gynecol.* 217, 200.e1-200.e17. 10.1016/j.ajog.2017.03.017 28347715

[BIO059326C18] Harmon, A. C., Cornelius, D. C., Amaral, L. M., Faulkner, J. L., Cunningham, M. W., Jr., Wallace, K. and LaMarca, B. (2016). The role of inflammation in the pathology of preeclampsia. *Clin. Sci.* 130, 409-419. 10.1042/CS20150702 PMC5484393 26846579

[BIO059326C19] Heyninck, K., De Valck, D., Vanden Berghe, W., Van Criekinge, W., Contreras, R., Fiers, W., Haegeman, G. and Beyaert, R. (1999). The zinc finger protein A20 inhibits TNF-induced NF-κB-dependent gene expression by interfering with an RIP- or TRAF2-mediated transactivation signal and directly binds to a novel NF-κB-inhibiting protein ABIN. *J. Cell Biol.* 145, 1471-1482. 10.1083/jcb.145.7.1471 10385526 PMC2133159

[BIO059326C20] Heyninck, K., Kreike, M. M. and Beyaert, R. (2003). Structure-function analysis of the A20-binding inhibitor of NF-κB activation, ABIN-1. *FEBS Lett.* 536, 135-140. 10.1016/S0014-5793(03)00041-3 12586352

[BIO059326C21] Koong, A. C., Chen, E. Y. and Giaccia, A. J. (1994). Hypoxia causes the activation of nuclear factor kappa B through the phosphorylation of I kappa B alpha on tyrosine residues. *Cancer Res.* 54, 1425-1430. 8137243

[BIO059326C22] Lemm, I., Girard, C., Kuhn, A. N., Watkins, N. J., Schneider, M., Bordonne, R. and Lührmann, R. (2006). Ongoing U snRNP biogenesis is required for the integrity of Cajal bodies. *Mol. Biol. Cell* 17, 3221-3231. 10.1091/mbc.e06-03-0247 16687569 PMC1483051

[BIO059326C23] Lett, K. E., Logan, M. K., McLaurin, D. M. and Hebert, M. D. (2021). Coilin enhances phosphorylation and stability of DGCR8 and promotes miRNA biogenesis. *Mol. Biol. Cell* 32, br4. 10.1091/mbc.E21-05-0225 34319763 PMC8684749

[BIO059326C24] Lingappan, K. (2018). NF-κB in oxidative stress. *Curr. Opin. Toxicol.* 7, 81-86. 10.1016/j.cotox.2017.11.002 29862377 PMC5978768

[BIO059326C25] Litang, Z., Hong, W., Weimin, Z., Xiaohui, T. and Qian, S. (2017). Serum NF-κBp65, TLR4 as biomarker for diagnosis of preeclampsia. *Open Med.* 12, 399-402. 10.1515/med-2017-0057 PMC5757355 29318184

[BIO059326C26] Liu, T., Zhang, L., Joo, D. and Sun, S. C. (2017). NF-κB signaling in inflammation. *Signal Transduct. Target. Ther.* 2, 17023. 10.1038/sigtrans.2017.23 29158945 PMC5661633

[BIO059326C27] Logan, M. K., Burke, M. F. and Hebert, M. D. (2018). Altered dynamics of scaRNA2 and scaRNA9 in response to stress correlates with disrupted nuclear organization. *Biol. Open* 7, bio037101. 10.1242/bio.037101 30177550 PMC6176948

[BIO059326C28] Logan, M. K., McLaurin, D. M. and Hebert, M. D. (2020). Synergistic interactions between Cajal bodies and the miRNA processing machinery. *Mol. Biol. Cell* 31, 1561-1569. 10.1091/mbc.E20-02-0144 32432989 PMC7521794

[BIO059326C29] Logan, M. K., Lett, K. E. and Hebert, M. D. (2021). The Cajal body protein Coilin is a regulator of the miR-210 hypoxamiR and influences MIR210HG alternative splicing. *J. Cell Sci.* 134, jcs258575. 10.1242/jcs.258575 34137440

[BIO059326C30] Madrid, L. V., Mayo, M. W., Reuther, J. Y. and Baldwin, A. S. Jr. (2001). Akt stimulates the transactivation potential of the RelA/p65 Subunit of NF-κB through utilization of the IκB kinase and activation of the mitogen-activated protein kinase p38. *J. Biol. Chem.* 276, 18934-18940. 10.1074/jbc.M101103200 11259436

[BIO059326C31] Maynard, S. E., Min, J.-Y., Merchan, J., Lim, K.-H., Li, J., Mondal, S., Libermann, T. A., Morgan, J. P., Sellke, F. W., Stillman, I. E. et al. (2003). Excess placental soluble fms-like tyrosine kinase 1 (sFlt1) may contribute to endothelial dysfunction, hypertension, and proteinuria in preeclampsia. *J. Clin. Invest.* 111, 649-658. 10.1172/JCI17189 12618519 PMC151901

[BIO059326C32] Maynard, S. E., Venkatesha, S., Thadhani, R. and Karumanchi, S. A. (2005). Soluble Fms-like tyrosine kinase 1 and endothelial dysfunction in the pathogenesis of preeclampsia. *Pediatr. Res.* 57, 1R-7R. 10.1203/01.PDR.0000159567.85157.B7 15817508

[BIO059326C33] Melvin, A., Mudie, S. and Rocha, S. (2011). Further insights into the mechanism of hypoxia-induced NFκB. *Cell Cycle* 10, 879-882. 10.4161/cc.10.6.14910 21325892 PMC3100871

[BIO059326C34] Nanda, S. K., Venigalla, R. K., Ordureau, A., Patterson-Kane, J. C., Powell, D. W., Toth, R., Arthur, J. S. and Cohen, P. (2011). Polyubiquitin binding to ABIN1 is required to prevent autoimmunity. *J. Exp. Med.* 208, 1215-1228. 10.1084/jem.20102177 21606507 PMC3173241

[BIO059326C35] O'Brien, M., Baczyk, D. and Kingdom, J. C. (2017). Endothelial dysfunction in severe preeclampsia is mediated by soluble factors, rather than extracellular vesicles. *Sci. Rep.* 7, 5887. 10.1038/s41598-017-06178-z 28725005 PMC5517616

[BIO059326C36] Olarerin-George, A. O., Anton, L., Hwang, Y. C., Elovitz, M. A. and Hogenesch, J. B. (2013). A functional genomics screen for microRNA regulators of NF-κB signaling. *BMC Biol.* 11, 19. 10.1186/1741-7007-11-19 23448136 PMC3621838

[BIO059326C37] Parrish, M. R., Murphy, S. R., Rutland, S., Wallace, K., Wenzel, K., Wallukat, G., Keiser, S., Ray, L. F., Dechend, R., Martin, J. N. et al. (2010). The effect of immune factors, tumor necrosis factor-alpha, and agonistic autoantibodies to the angiotensin II type I receptor on soluble fms-like tyrosine-1 and soluble endoglin production in response to hypertension during pregnancy. *Am. J. Hypertens.* 23, 911-916. 10.1038/ajh.2010.70 20431529 PMC3500852

[BIO059326C38] Poole, A. R., Enwerem, I. I., Vicino, I. A., Coole, J. B., Smith, S. V. and Hebert, M. D. (2016). Identification of processing elements and interactors implicate SMN, coilin and the pseudogene-encoded coilp1 in telomerase and box C/D scaRNP biogenesis. *RNA Biol.* 13, 955-972. 10.1080/15476286.2016.1211224 27419845 PMC5056767

[BIO059326C39] Qi, J. W., Qin, T. T., Xu, L. X., Zhang, K., Yang, G. L., Li, J., Xiao, H. Y., Zhang, Z. S. and Li, L. Y. (2013). TNFSF15 inhibits vasculogenesis by regulating relative levels of membrane-bound and soluble isoforms of VEGF receptor 1. *Proc. Natl. Acad. Sci. USA* 110, 13863-13868. 10.1073/pnas.1304529110 23918400 PMC3752234

[BIO059326C40] Qu, L., Ji, Y., Zhu, X. and Zheng, X. (2015). hCINAP negatively regulates NF-κB signaling by recruiting the phosphatase PP1 to deactivate IKK complex. *J. Mol. Cell Biol.* 7, 529-542. 10.1093/jmcb/mjv041 26089539

[BIO059326C41] Rajakumar, A., Powers, R. W., Hubel, C. A., Shibata, E., von Versen-Höynck, F., Plymire, D. and Jeyabalan, A. (2009). Novel soluble Flt-1 isoforms in plasma and cultured placental explants from normotensive pregnant and preeclamptic women. *Placenta* 30, 25-34. 10.1016/j.placenta.2008.10.006 19010535 PMC2607481

[BIO059326C42] Sakurai, H., Chiba, H., Miyoshi, H., Sugita, T. and Toriumi, W. (1999). IκB kinases phosphorylate NF-κB p65 subunit on serine 536 in the transactivation domain. *J. Biol. Chem.* 274, 30353-30356. 10.1074/jbc.274.43.30353 10521409

[BIO059326C43] Sánchez-Aranguren, L. C., Prada, C. E., Riaño-Medina, C. E. and Lopez, M. (2014). Endothelial dysfunction and preeclampsia: role of oxidative stress. *Front. Physiol.* 5, 372. 10.3389/fphys.2014.00372 25346691 PMC4193194

[BIO059326C44] Santama, N., Ogg, S. C., Malekkou, A., Zographos, S. E., Weis, K. and Lamond, A. I. (2005). Characterization of hCINAP, a novel coilin-interacting protein encoded by a transcript from the transcription factor TAFIID32 locus. *J. Biol. Chem.* 280, 36429-36441. 10.1074/jbc.M501982200 16079131

[BIO059326C45] Sasaki, C. Y., Barberi, T. J., Ghosh, P. and Longo, D. L. (2005). Phosphorylation of RelA/p65 on serine 536 defines an IκBα-independent NF-κB pathway. *J. Biol. Chem.* 280, 34538-34547. 10.1074/jbc.M504943200 16105840

[BIO059326C46] Shah, T. J. and Walsh, S. W. (2007). Activation of NF-kappaB and expression of COX-2 in association with neutrophil infiltration in systemic vascular tissue of women with preeclampsia. *Am. J. Obstet. Gynecol.* 196, 48.e1-48.e8. 10.1016/j.ajog.2006.08.038 17240230

[BIO059326C47] Shih, D. Q., Kwan, L. Y., Chavez, V., Cohavy, O., Gonsky, R., Chang, E. Y., Chang, C., Elson, C. O. and Targan, S. R. (2009). Microbial induction of inflammatory bowel disease associated gene TL1A (TNFSF15) in antigen presenting cells. *Eur. J. Immunol.* 39, 3239-3250. 10.1002/eji.200839087 19839006 PMC2839414

[BIO059326C48] Song, Y. J., Jen, K. Y., Soni, V., Kieff, E. and Cahir-McFarland, E. (2006). IL-1 receptor-associated kinase 1 is critical for latent membrane protein 1-induced p65/RelA serine 536 phosphorylation and NF-κB activation. *Proc. Natl. Acad. Sci. USA* 103, 2689-2694. 10.1073/pnas.0511096103 16477006 PMC1413826

[BIO059326C49] Taganov, K. D., Boldin, M. P. and Baltimore, D. (2007). MicroRNAs and immunity: tiny players in a big field. *Immunity* 26, 133-137. 10.1016/j.immuni.2007.02.005 17307699

[BIO059326C50] Takeuchi, H., Hirano, T., Whitmore, S. E., Morisaki, I., Amano, A. and Lamont, R. J. (2013). The serine phosphatase SerB of Porphyromonas gingivalis suppresses IL-8 production by dephosphorylation of NF-κB RelA/p65. *PLoS Pathog.* 9, e1003326. 10.1371/journal.ppat.1003326 23637609 PMC3630210

[BIO059326C51] Torchinsky, A. and Toder, V. (2004). To die or not to die: the function of the transcription factor NF-κB in embryos exposed to stress. *Am. J. Reprod. Immunol.* 51, 138-143. 10.1046/j.8755-8920.2003.00134.x 14748840

[BIO059326C52] Vaughan, J. E. and Walsh, S. W. (2012). Activation of NF-κB in placentas of women with preeclampsia. *Hypertens. Pregnancy* 31, 243-251. 10.3109/10641955.2011.642436 22380486 PMC3542769

[BIO059326C53] Velma, V., Carrero, Z. I., Cosman, A. M. and Hebert, M. D. (2010). Coilin interacts with Ku proteins and inhibits in vitro non-homologous DNA end joining. *FEBS Lett.* 584, 4735-4739. 10.1016/j.febslet.2010.11.004 21070772 PMC3000556

[BIO059326C54] Verstrepen, L., Carpentier, I., Verhelst, K. and Beyaert, R. (2009). ABINs: A20 binding inhibitors of NF-κB and apoptosis signaling. *Biochem. Pharmacol.* 78, 105-114. 10.1016/j.bcp.2009.02.009 19464428

[BIO059326C55] Yue, T. L., Ni, J., Romanic, A. M., Gu, J. L., Keller, P., Wang, C., Kumar, S., Yu, G. L., Hart, T. K., Wang, X. et al. (1999). TL1, a novel tumor necrosis factor-like cytokine, induces apoptosis in endothelial cells. Involvement of activation of stress protein kinases (stress-activated protein kinase and p38 mitogen-activated protein kinase) and caspase-3-like protease*. *J. Biol. Chem.* 274, 1479-1486. 10.1074/jbc.274.3.1479 9880523

[BIO059326C56] Zaqout, S., Becker, L. L. and Kaindl, A. M. (2020). Immunofluorescence staining of paraffin sections step by step. *Front. Neuroanat.* 14, 582218. 10.3389/fnana.2020.582218 33240048 PMC7680859

[BIO059326C57] Zhai, Y., Yu, J., Iruela-Arispe, L., Huang, W.-Q., Wang, Z., Hayes, A. J., Lu, J., Jiang, G., Rojas, L., Lippman, M. E. et al. (1999). Inhibition of angiogenesis and breast cancer xenograft tumor growth by VEGI, a novel cytokine of the TNF superfamily. *Int. J. Cancer* 82, 131-136.<131::AID-IJC22>3.0.CO;2-O 10360832 10.1002/(sici)1097-0215(19990702)82:1<131::aid-ijc22>3.0.co;2-o

[BIO059326C58] Zhou, A., Scoggin, S., Gaynor, R. B. and Williams, N. S. (2003). Identification of NF-κB-regulated genes induced by TNFα utilizing expression profiling and RNA interference. *Oncogene* 22, 2054-2064. 10.1038/sj.onc.1206262 12673210

