# Supplementary material for: Coilin as a regulator of NF-kB mediated inflammation in preeclampsia
Source: Biol Open. 2022 Jul 25;11(7):bio059326. doi: 10.1242/bio.059326 (PMC9346287; doi:10.1242/bio.059326)
Supplement: Supplementary information [file biolopen-11-059326-s1.pdf]

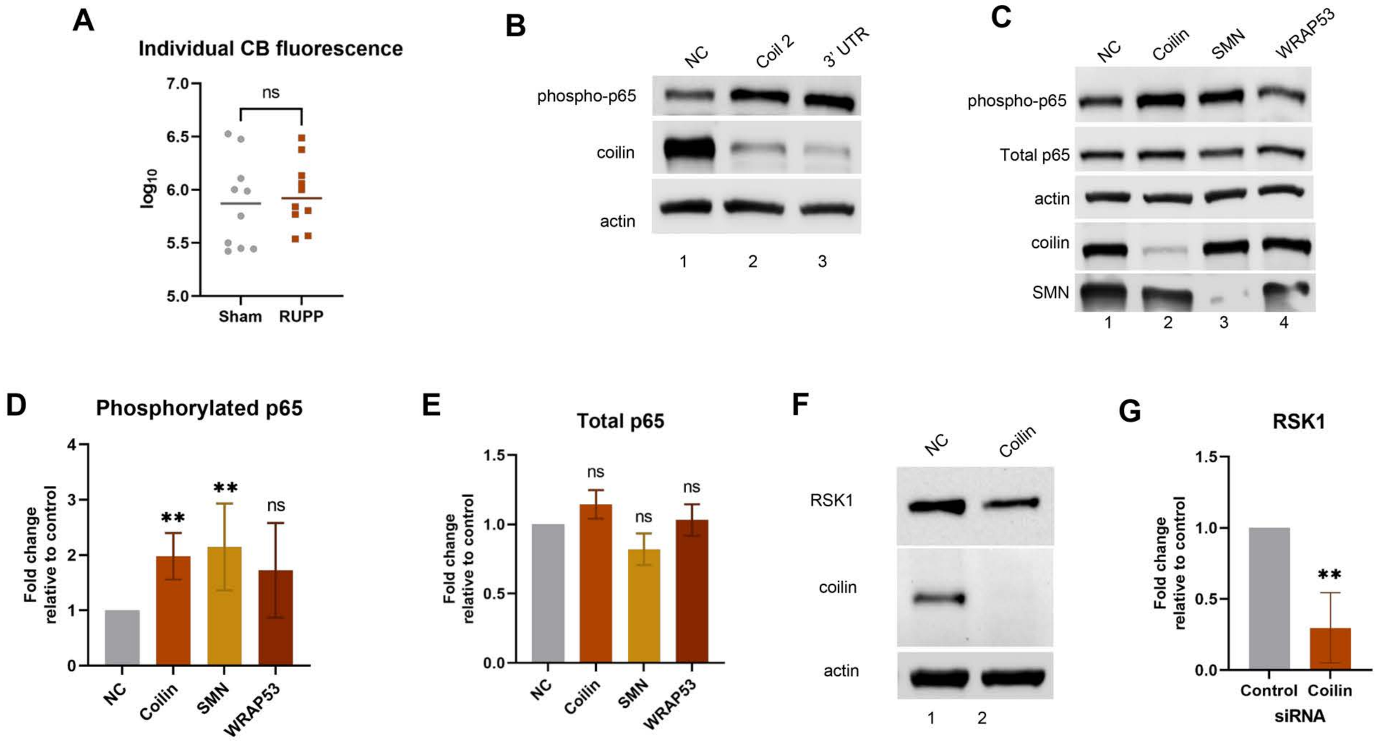

Supplemental Figure 1

Fig. S1. (A) Histogram depicting individual CB (coilin) fluorescence from Sham and RUPP rat trophoblasts on a log10 scale. N=275. **(B)** Normoxic JEG-3 cells were transfected with negative control and 2 different coilin siRNAs (coil 2 and coil 3'UTR) for 72 hrs to confirm the increase in phosphorylated p65 seen with coilin knockdown. **(C)** Normoxic JEG-3 cells were transfected with negative control, coilin, SMN, and WRAP53 siRNAs for 72 hrs to establish if the KD of other CB-enriched proteins alter phosphorylated p65 levels. **(D and E)** Phosphorylated p65 and total p65 signals were quantified by normalizing to β-actin relative to that obtained with the negative control siRNA. N=3 biologicals. **(F)** JEG-3 cells were transfected with control or coilin siRNA as described in (B) to assess RSK1 protein levels with coilin knockdown. **(G)** RSK1 signal in coilin KD lysate was quantified by normalizing to β-actin relative to that obtained with control siRNA, which is set to 1. For all panels with statistics, error bars represent standard deviation and ** = $p < 0.005$.

Biology Open • Supplementary information

Table S1.

sFLT1-i13	Forward: CTGCAGAGCCAGGAATGTATACACAGG<br>Reverse: CGAGCCTGAAAGTTAGCAACAGTG
FLT1	Forward: GCATACCTCACTGTTCAAGGAACC<br>Reverse: CAGCCACACAGGTGCATGTTAG
TNIP1	Forward: GCGCCTAGTGAAGGAGAATTCC<br>Reverse: CTGGTGAATTCTGCTCCTCAGG
TNFSF15	Forward: GCACACCTGACAGTTGTGAGAC<br>Reverse: CACAGTGATGGAGTCTGGCTTG
